# The impact of COVID-19 on memory: Recognition for masked and unmasked faces

**DOI:** 10.3389/fpsyg.2022.960941

**Published:** 2022-10-06

**Authors:** Natália Guerra, Raquel Pinto, Pedro S. Mendes, Pedro F. S. Rodrigues, Pedro B. Albuquerque

**Affiliations:** ^1^School of Psychology, University of Minho, Braga, Portugal; ^2^Centro de Investigação e Intervenção Social, Instituto Universitário de Lisboa (ISCTE-IUL), Lisbon, Portugal; ^3^Portucalense Institute for Human Development (INPP), Portucalense University, Porto, Portugal

**Keywords:** COVID-19, surgical masks, faces, memory, recognition

## Abstract

Considering the current state of the worldwide pandemic, it is still common to encounter people wearing face protection masks. Although a safety measure against COVID-19, face masks might be compromising our capacity for face recognition. We conducted an online study where 140 participants observed masked and unmasked faces in a within-subjects design and then performed a recognition memory task. The best performance was found when there were no masks either at study and test phase, i.e., at the congruent unmasked condition. The worst performance was found for faces encoded with a mask but tested without it (i.e., masked-unmasked incongruent condition), which can be explained by the disruption in holistic face processing and the violation of the encoding specificity principle. Interestingly, considering the unmasked-masked incongruent condition, performance was probably affected by the violation of the encoding specificity principle but protected by holistic processing that occurred during encoding.

## Introduction

In June 2020, the World Health Organization (WHO) recommended the use of face masks in public places to prevent the spread of COVID-19 (World Health Organization, 2020). Although the use of masks is no longer mandatory in some countries, as in the case of Portugal (Decree-Law 30-E/2022, 2022), many people continue to use them as a way to prevent infections, particularly in public transport and health institutions. Despite its effectiveness in reducing virus transmission (Howard et al., 2021), the use of face masks may impair people’s ability to recognize facial identity and expressions since face masks cover about 60–70% of the face area that is relevant for the identification of a person’s identity (e.g., Tsao and Livingstone, 2008), emotional state and for judgments of friendliness or attractiveness (Goldstein and Brockmole, 2017), hence hampering social interactions (e.g., Mheidly et al., 2020).

Faces seem to be processed holistically (Farah et al., 1998; Taubert et al., 2011; Logan et al., 2017; Meltzer and Bartlett, 2019), meaning they are perceived as a whole rather than a single combination of each component (e.g., eyes, nose, mouth). When this holistic processing is disrupted, face and emotion identification is affected (Kret and de Gelder, 2012; Curby et al., 2013), and memory for faces may be impaired (McKelvie, 1976; Manley et al., 2019).

The face inversion effect is an example of disruption in holistic face processing. When faces are presented upside-down, they take longer to process than other inverted stimuli, suggesting that disrupting the usual way we perceive faces (i.e., upright) affects their holistic processing (e.g., Taubert et al., 2011). Another possible way to disrupt holistic face processing is by partially occluding facial features, such as wearing sunglasses or headwear. In a study exploring face occlusion by different headdresses, Kret and de Gelder (2012) found that happy and sad expressions were harder to identify when faces were covered by a niqab (i.e., a garment of clothing that covers the face but leaves the eyes visible), but fear was still easily recognizable. Such findings are in line with a negative bias in perceiving the emotions of faces covered in niqabs, in which people attributed lower ratings of happiness when faces covered by a niqab depicted happiness (Fischer et al., 2012). One possible explanation for these changes in perceived emotion might relate to the different contributions of upper and lower facial features for emotion recognition.

There is plenty of support for the distinct contribution of upper and lower components of faces to emotional perception. For example, mouth movements seem to play a significant role in both static and dynamic facial expressions (Blais et al., 2012), and in a study recurring to eye-tracking, it was found that people spent more time looking (i.e., higher fixations) at the eyes for sadness and the mouth for happiness (Eisenbarth and Alpers, 2011). Additionally, happiness and disgust seem to be emotions promptly identified by observing the mouth region. Still, other emotions such as anger, sadness, and fear can be successfully recognized by relying on information from the area of the eyes (Wegrzyn et al., 2017). While showing that compromising holistic processing affects the recognition of facial expressions and emotions, these studies also provide evidence that some facial features can be informative on their own.

There might be cases in which a single facial feature relates to a specific emotion and is sufficient to identify it, such as the relation between mouth and happiness. Calvo and Nummenmaa (2008) found that in a visual search task, where emotional faces should be identified among neutral faces, the inversion of the faces delayed the detection of fearful, angry, and sad expressions, but not happy, surprised, and disgusted faces in comparison with an upright condition. The authors argued that the ability to detect fear, anger, and sadness might rely more on holistic processing (Calvo and Nummenmaa, 2008).

In addition to emotional processing, facial recognition is also impaired when specific facial features are compromised. For example, Sadr et al. (2003) found that removing the eyebrows from familiar faces significantly impaired recognition. Also, Abudarham and Yovel (2019) observed that distances and general shapes, such as eye distance and face proportion were less critical for face identification, thus reinforcing the importance of crucial facial parts for the identification of faces. Hence, even when a single facial feature is connected to recognizing a specific trait or emotion, the overall body of evidence suggests that impairing the holistic processing of faces will likely impair their recognition. Other examples of impaired face recognition were observed when faces changed hairstyle or eyeglasses were added or removed (Righi et al., 2012) and when faces had sunglasses covering the eyes or bandanas occluding the lower part of the face (Nguyen and Pezdek, 2017), hinting that face coverings affect people’s ability to recognize a face.

When using face masks to prevent the dissemination of COVID-19, face processing is probably impaired since such masks cover the lower facial structures (i.e., mouth, nose, cheeks, and chin), which will disrupt the holistic processing of the face and will not allow observing parts of the face that can be crucial for recognizing facial expressions (e.g., Carbon, 2020; Calbi et al., 2021). A recent review compiled evidence suggesting that wearing face masks affects how faces are processed in the COVID-19 pandemic context (see Pavlova and Sokolov, 2022). However, most studies have focused on the emotional aspects of face processing. For example, it was found that emotions that rely heavily on mouth movements to be identified (e.g., sadness, happiness, and anger) were often misinterpreted as neutral (Carbon, 2020), but yet it seems that people rate happy masked faces more positively than angry and neutral ones (Calbi et al., 2021). Moreover, face masks seem to reduce perceived emotion expression intensity and impact warm and pleasant interactions, that is, facial mimicry of happy expressions (Kastendieck et al., 2021). Additionally, covering unattractive faces with masks increased perceived attractiveness (Patel et al., 2020). While these studies explored the emotions and attractiveness of masked faces, other studies tried to explore different dimensions of face processing in the presence of a mask amidst a pandemic, namely, face recognition.

Carragher and Hancock (2020) found that masks impaired face recognition when they asked participants to judge whether two presented face photographs showed the same person. Faces were presented in three conditions, namely, both faces without masks (control), one of the faces wearing a mask (mixed), or both faces wearing masks (masked). Their results revealed high error rates for face matching performance in mixed and masked conditions, suggesting an impairment in face perception (Carragher and Hancock, 2020), further hinting that face recognition could also be affected. Furthermore, there was evidence for a familiarity effect when assessing masked faces, with participants biased to report familiar faces as “matches” and unfamiliar faces as “mismatches”. These findings strongly suggest that face recognition is affected when wearing masks. However, Carragher and Hancock’s (2020) study used a face-matching task, which differs test (old vs. new).

Additionally, Freud et al. (2020, 2021) explored whether wearing face masks would impair face recognition by disrupting holistic processing. In their study (2020), participants completed the Cambridge Face Memory Test—a widely used test of face recognition abilities—with upright and inverted faces in one of two conditions: presented faces either had or did not have a mask, both at the encoding and retrieval phase. Results revealed a robust decrease in face recognition performance for masked faces compared to non-masked faces. Additionally, the authors observed a significant reduction of the face inversion effect for masked faces, suggesting that participants must have relied on the available features instead of the holistic processing to recognize faces. In Experiment 2, Freud et al. (2020) extended these findings to situations in which the masked faces appeared only at encoding (i.e., study phase) or only at retrieval (i.e., test phase). Results suggested that face processing and recognition are highly susceptible to the inclusion of masks. Critically, Freud et al. (2020, 2021) and Carragher and Hancock (2020) used a between-subjects design, so it is unclear whether recognition for masked faces is impaired compared to unmasked faces within-subjects design.

There is scarce previous evidence hinting how the presence of a mask may impair the recognition memory for faces in a within-subjects design, albeit in a non-pandemic context. Namely, a study examined the role of masked-face lineups in eyewitness identification of a masked person (Manley et al., 2019). Manley et al. (2019, Exp.2) found that participants’ recognition performance was the highest when encoding a full face (unmasked face at the study phase) and with a full-face lineup (unmasked face at the test phase) but was the worst after encoding a face with a ski mask (masked face at study phase) and with a full-face lineup (unmasked face at test phase). Results showed that matching the conditions (specifically, the use or not of a ski mask) in the study and test phases may enhance eyewitness identification accuracy. These results could be based on the transfer-appropriate processing framework, meaning that the performance on a memory test is higher when the processes activated at retrieval match those at encoding (e.g., Morris et al., 1977). This pattern of results was observed in another study that presented unmasked faces in the test phase. These faces were presented previously, in a between-design, with standard surgical masks, transparent masks, or without masks. Unmasked faces were identified better when previously shown without masks than those presented with transparent or standard masks. No differences were found between the two types of masks, indicating that both equally impair the subsequent identification of the face (Marini et al., 2021). This last result also shows that face identification depends not only on the mouth region (uncovered with the transparent mask) but also on additional cues of the lower half of the face, such as the jaw and cheeks, which are covered by both types of masks.

Here, we aimed to test recognition memory for masked and unmasked faces using a within-subjects design. To this end, we manipulated the presence of a mask during the study and test phases, which allowed us to see whether congruence between both phases (masked-masked and unmasked-unmasked faces) would lead to better facial recognition performance. We expected that the presence of a mask would impair recognition memory performance regardless of the mask being presented at encoding or retrieval. We also expected recognition memory performance to be the worst for faces wearing a mask at encoding but not at the test phase. Finally, we expected better facial recognition when congruence between the study and the test phase (masked-masked and unmasked-unmasked) occurs than when there is incongruence (masked-unmasked and unmasked-masked). Given the safety measures imposed during this period, the procedure was carried out entirely online.

## Methods

### Participants

One hundred and forty undergraduate students agreed to participate in our study (78 females; *M*_*age*_ = 20.74, *SD* = 3.13), receiving course credits for their participation. One participant was excluded from the initial sample because she did not complete the distractive task. Recruitment of participants was conducted through an online course-credit platform of the University of Minho. The sample size was calculated *a priori* through G*Power3.1.9.6 (Faul et al., 2007), targeting a bi-factorial repeated-measures ANOVA, suggesting a minimum of 36 participants to detect a medium effect size (*f* = 0.25), given an alpha (α) of 0.05 and a statistical power of 0.95. The local Ethics Committee approved this research. Data were collected during the pandemic (from December 2020 to November 2021), when face masks were mandatory in all regions of Portugal (Decree-Law 20/2020, 2020).

### Materials

The experimental procedure was created using Qualtrics XM (Provo, UT) (Qualtrics, 2021). Forty-eight neutral Caucasian faces (24 male; 24 female) were selected from the free-access “Chicago Face Database” (Ma et al., 2015). We decided to apply several criteria to their selection concerning the faces used, namely, estimated age 18–26 years old (*M* = 23.82, *SD* = 1.81) and masculinity and femininity judgment above 98%. Since our goal was not to explore the role of emotion in face recognition, we opted for a neutral face expression by selecting faces whose emotional expressions were evaluated below 3.5 on a 7-point Likert scale (1 = not at all; 7 = extremely). The average evaluation for emotional expressions were the following: afraid = 2.04; angry = 2.37; disgusted = 2.10; happy = 2.28; sad = 2.61; and surprised = 1.66.

**FIGURE 1:**
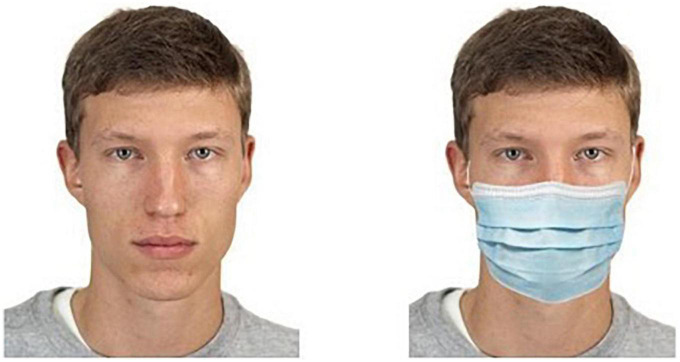
Example of an original photo and its manipulated version with a superimposed mask. Reproduced with permission from the Chicago Face Database (Ma et al., 2015, p. 1125).

Every photo was duplicated, and one of the items was then manipulated with Adobe Photoshop CC (Adobe Systems Incorporated, 2014) by superimposing a standard surgical mask on the original photo, a technique usually used in other studies (e.g., Carragher and Hancock, 2020). All the process was done so that such images would appear as authentic as possible. In the end, all the 48 faces had two versions: unmasked (original photo) and masked (manipulated photo). See Figure 1 for an example.

### Procedure

As mentioned, the study was conducted entirely online. Firstly, informed consent was obtained from participants, and a sociodemographic questionnaire was completed. The main procedure included two phases: a study and a test phase. Before the study phase, participants were instructed to be attentive while studying masked and unmasked faces, given that later they would have a recognition test. During the study phase, 32 of the 48 faces selected for this study, matched by sex, were randomly presented, half depicting masked faces and the other half unmasked faces (the 32 faces were counterbalanced across participants so that each face was presented in its two versions, masked and unmasked, in both study and test phase). Each face was displayed for 3 s with a blank screen interval of 2 s between them.

Following the study phase, a distractive task was presented for 3 min. In a verbal fluency task, the participants had to type in as many category examples as possible for 30 s (e.g., Body Parts, European Countries, Fruits, Colors, Furniture Items). The categories were presented individually and consecutively, with a 5-s interval between them. Responses to this task were not analyzed but were checked for completion, and as mentioned before, one participant was excluded because she did not complete the task.

**FIGURE 2:**
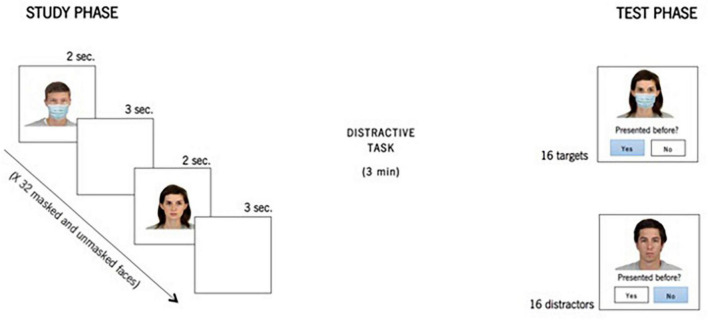
Illustrative scheme of stimuli presented in the study and test phases. Reproduced with permission from the Chicago Face Database (Ma et al., 2015, p. 1125).

Thirty-two images were presented in the test phase, 16 of them as targets and the other 16 as distractors. The distractors were balanced according to the presence or absence of a mask. Of the 16 targets, half were previously presented with a mask to participants, and the other half were unmasked. In summary, four faces appeared with a mask in both moments (masked-masked); four faces were presented unmasked in the two phases (unmasked-unmasked); four faces appeared in the study phase without a mask but in the test phase with a superimposed mask (unmasked-masked); and, finally, four faces were exposed in the study phase with a mask but in the test phase without it (masked-unmasked). See Figure 2 for an illustrative scheme of the stimuli manipulation and presentation. The stimuli were randomly presented, and for each stimulus, two response options were displayed: “Yes, this face was presented before” and “No, this face was not presented before.” Participants judged whether that face had been presented before or if it was the first time they had seen it by using their computer mouse. The procedure took approximately 20 min to complete.

### Design and analyses

We applied a 2 (face at study: masked vs. unmasked) × 2 (face at test: masked vs. unmasked) within-subjects design. Face recognition performance was measured through the sensitivity index or d-prime. A d-prime (d′) score [z (Hits) – z (False Alarms)] was calculated for the recognition memory results on each condition of the bi-factorial also calculated response bias (c) score [–(z (Hits) + z (False Alarms))/2]. Hits refer to “yes” responses to the faces that were presented in the study phase (correct “yes” response), and false alarms refer to “yes” responses to faces that were not presented at the study phase (incorrect “yes” responses).

The software used for the data analysis was JASP 0.15 (JASP Team, 2021). To explore the effect of wearing a mask on face recognition, a 2 (face at study: masked vs. unmasked) × 2 (face at test: masked vs. unmasked) repeated-measures ANOVA was performed, and all prerequisites were verified. Additionally, we ran a linear mixed model (LMM) analysis in RStudio (version 2021.09.1 + 372) using the lme4 package (Bates et al., 2015). We included the d-prime (d′) score and response bias (c) score as the dependent variables, and we included fixed effects of mask-at-study and mask-at-test, as well as the interaction between the two. Also, we added participants as a cluster variable with random intercept (1| participant) and random slope (mask| participant). As for significance, it was calculated recurring to the lmerTest package (Kuznetsova et al., 2017), and pairwise comparisons were performed running the emmeans package (Lenth et al., 2022). The models’ specifications were as follows: d ∼ Mask_Study*Mask_Test + (Mask_Study| Participant) + (Mask_Test| Participant) + (1| Participant) and c ∼ Mask_Study*Mask_Test + (Mask_Study| Participant) + (Mask_Test| Participant) + (1| Participant).

## Results

**FIGURE 3:**
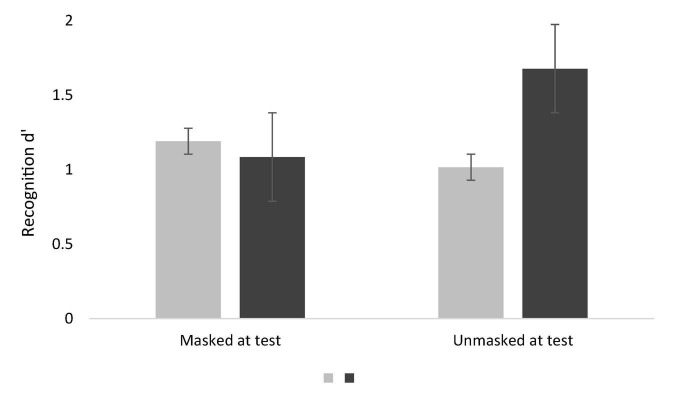
Mean d′ scores for masked and unmasked faces at study and test phase. The error bars correspond to the SEM (Standard Error of the Mean). Grey corresponds to masked at study and black to unmasked at study.

Firstly, we present results obtained through repeated-measures ANOVA. Concerning *d*′, a significant main effect of face at study was found, *F*(1, 139) = 34.15, *p* < 0.001, η_p_^2^ = 0.20, revealing a better recognition for unmasked faces (*M* = 1.38; *SE* = 0.06) compared to masked faces at study (*M* = 1.10; *SE* = 0.06). A main effect of face at test was also found, *F*(1, 139) = 8.88, *p* = 0.003, η_p_^2^ = 0.06, showing a better recognition for faces unmasked at test (*M* = 1.35; *SE* = 0.06) than masked faces (*M* = 1.14; *SE* = 0.06). Also, we found a significant interaction, *F*(1, 139) = 53.07, *p* < 0.001, η_p_^2^ = 0.28. *Post hoc* tests with Bonferroni correction revealed a significantly better recognition of faces presented without a mask in both phases (all *p* < 0.001) compared to other conditions (for descriptive values, see Figure 3). The differences between the remaining conditions were not significant (all *p* > 0.286). Our results suggest that face recognition was maximized when faces were presented unmasked at study and test, the pre-pandemic everyday face processing and recognition. Results also showed that face recognition was impaired when faces were presented with masks, whether at encoding, recognition, or both moments.

Considering the analysis of response bias (*c*) a significant main effect of face at study was found, *F*(1, 139) = 34.15, *p* < 0.001, η_p_^2^ = 0.20, revealing a more conservative criteria for the recognition of masked faces (*M* = 0.14; *SE* = 0.03) compared to unmasked faces at study (*M* = 0.003; *SE* = 0.03). A significant main effect of face at test was also found, *F*(1, 139 = 49.53, *p* ≤ 0.001, η_p_^2^ = 0.26, showing a higher *c* value for unmasked faces (*M* = 0.22; *SE* = 0.03) than for masked faces at test (*M* = −0.07; *SE* = 0.03). Finally, we found a significant interaction between both factors, *F*(1, 139) = 53.08, *p* < 0.001, η_p_^2^ = 0.28. *Post hoc* tests with Bonferroni correction revealed a significantly higher *c* value for the condition where the faces were presented with masks at study and unmasked at test (all *p* < 0.088), expressing a more conservative answer at recognition of such faces. Unmasked faces at both phases (*c* = 0.06) differed (*p* = 0.008) also from masked faces in both phases (*c* = −0.10). No other comparisons were significant (all *p* > 0.233).

A similar pattern of results was obtained with LMM. In terms of d′ score, there was a significant main effect for face at study (*beta* = −0.66, *t* = −9.32, *p* < 0.001), as well as for face at test (*beta* = −0.59, *t* = −6.87, *p* < 0.001). Moreover, a face at study by face at test interaction was also significant (*beta* = 0.76, *t* = 7.65, *p* < 0.001). Pairwise comparisons were performed, and, again, participants recognized better unmasked faces in both phases (all *p* < 0.001), while differences between the remaining conditions were non-significant (all *p* ≥ 0.182). Furthermore, in terms of c score a significant main effect of face at study was verified (*beta* = 0.33, *t* = 9.33, *p* < 0.001) as well as a main effect of face at test (*beta* = −0.10, *t* = −2.11, *p* = 0.04), once more the interaction between the two fixed effects was significant (*beta* = −0.38, *t* = −7.73, *p* < 0.001). Pairwise comparisons revealed that participants were more conservative while recognizing masked faces (*p* = 0.006) than unmasked faces at both phases. At the same time, the condition of masked faces at study differed from the unmasked at the test (all *p* < 0.001). The remaining comparisons were not statistically significant (all *p* > 0.15). Altogether, these results indicate that recognition performance was influenced by the presence of a mask either at the study or test phases. A copy of the data is available at the OSF Platform,^1^ and the R code can be requested from the corresponding author.

Additionally, a paired-sample *t*-test was conducted to verify whether there were any differences in face recognition performance when the presence or absence of mask at the two phases (i.e., study and test) were congruent (masked-masked, unmasked-unmasked) and incongruent (masked-unmasked, unmasked-masked). The analysis showed a significant difference in terms of face recognition between the two conditions (i.e., congruence and incongruence), *t*(139) = 7.47, *p* < 0.001, Cohen’s *d* = 0.63, 95 % CI [0.45, 0.81]. The results revealed higher ratings when there was congruence between the study phase and the test phase (*M* = 1.63; *SD* = 0.75) contrarily to the incongruence condition (*M* = 1.17; *SD* = 0.70).

## Discussion

The present work aimed to understand whether using face masks would impair people’s ability to recognize faces. To this end, we conducted an experiment on recognition memory for faces (i.e., both masked and unmasked) recurring to a within-subject design. To our knowledge, just another one has done it (Garcia-Marques et al., 2022). We expected to encounter some new data that could explain the difficulties one might have while interacting with a masked person, given the pandemic situation that is still ongoing.

As one might expect, we found an increased face recognition performance when participants observed unmasked faces both in the study and in the test phase since no constraints are imposed on our face recognition capacity in this condition, so there is a clear advantage for unmasked faces. On the other hand, results revealed that face recognition was impaired when faces were presented with masks, whether at study, at test, or in both phases. These results are in line with those obtained by Freud et al. (2020), in which the inclusion of a face mask led to an impaired performance regardless of whether the mask was included in the test or study phase.

And so, in agreement with our hypothesis, when comparing the study and the test phase, a significant main effect of *face at study* was found. Thus, revealing a better recognition of faces encoded without a mask. Equally, a significant main effect of *face at test* was also observed, indicating that unmasked faces were better recognized at this stage. Seemingly, to what Manley et al. (2019) reported, participants cannot engage with holistic processing when presented with masked faces during the study. Instead, they establish a feature-based encoding mechanism for the faces presented, but the recognition memory performance decays despite that. We had hypothesized this scenario.

Moreover, we have also found a significant main effect of face at test. It seems that participants’ recognition memory capacity was impaired mainly by the presence of masked faces during the recognition memory test. At this point, we could argue that our results agree with what we had anticipated.

Nonetheless, we also expected that recognition memory performance would be worse for faces wearing a mask at encoding but not on the test phase, a result observed by Manley et al. (2019), which examined the role of masked-face lineups in the eyewitness identification of a masked person. A similar result was also reported by Marini et al. (2021) in a study that examined the impact of the use of standard surgical masks and transparent masks in a between-subjects design. Unmasked faces were identified better when previously presented without masks compared to faces previously presented with transparent or standard masks. Previous research on eyewitness testimony suggested that a disguising element, such as eyeglasses encoded as part of the face, facilitate face recognition when the disguise remains at the lineup implementation (Righi et al., 2012). Considering these results, it can be expected that in the mask-at-study and mask-at-test condition the recognition will be better, although not significantly, than the face was encoded with a mask and tested without, hinting at an encoding impairment.

In addition to the affected holistic processing, a violation of the encoding specificity (ESP) principle occurs in this case. The ESP states that the conditions presented at encoding (e.g., masked faces) when disrupted at retrieval (e.g., unmasked faces) will lead to a significant impairment of memory performance (Tulving and Thomson, 1973). This principle is also verified by our results in the congruent conditions (masked-masked or unmasked-unmasked). Increased memory performance was confirmed in the congruent conditions, so ESP facilitates face recognition processes.

Albeit the results in the masked-unmasked condition, when the directly opposite situation occurred (i.e., unmasked face at study and masked at test), the disruption performance did not suffer a decay to the same extent. In this latter situation, although a violation of the encoding specificity principle occurred, the faces in the study phase were presented without masks, which allowed the holistic processing of the faces.

Therefore, if one has met certain people without them wearing a mask it could be easier to recognize them later since we held previous information of a “complete face version.” So, although there is no congruence between the phases, a masked face in the test phase can benefit from previous holistic processing. Previous literature has demonstrated that the holistic processing of faces favors their later recognition, even when faces might be submitted to certain deviations from their original form (Richler et al., 2011). Perhaps the same can be valid for faces partially occluded by masks.

In addition, regarding the response bias (c) score, participants seem to be more conservative (i.e., higher values) when recognizing masked faces. Likely, the presence of a mask left participants more uncertain, so they took fewer risks when recognizing faces with masks. Measuring response times in more controlled settings could be helpful in future studies.

Albeit previous studies have come forward with the evidence that observing masked faces imposes a greater challenge to face recognition, with our approach, we were able to find the particular aspect of the unmasked-masked incongruent condition. For this case, we can now consider the possibility of a beneficial effect of holistic processing that can be transferred from encoding to retrieval. For instance, if we consider the incongruent masked-unmasked condition, in which there is no opportunity for holistic processing to occur during encoding, participants’ performance seems to be hampered to a greater degree. In the masked-unmasked condition, while the participants observed masked faces (study phase) and tested with unmasked forms of those faces, they encountered half of faces that they had never holistically processed before. Previous literature explains this occurrence due to feature-based processing (Manley et al., 2019).

Our study emphasizes the role of holistic processing in face recognition, considering that it might potentiate face recognition even under extremely disruptive circumstances, i.e., occlusion of a face’s lower half. Taken together, our results reveal that using a face mask hampers face recognition, especially if there is no congruence between the encoding and retrieval conditions. However, we assume that if holistic processing is allowed during encoding, the detrimental effects caused by mask use (i.e., disruption) on face recognition can be mitigated to a certain degree. In other words, allowing a face to be processed holistically will be crucial to overcoming the difficulties imposed by using a mask (i.e., violation of the encoding specificity principle or congruence), although not completely. Practically, this implies that if a face is first processed holistically, it will stand a better chance of being correctly recognized even when covered by a mask.

Human beings are well trained to recognize unmasked faces (i.e., holistic processing) as a result of adaptive processes occurring throughout the evolution of our species. It would be interesting if future studies took a longitudinal approach to understand if we can adapt and improve our capability to recognize masked faces (i.e., feature-based processing). It has already been proven that the cultural context we are inserted in plays a huge part in determining how accurately we can identify emotions (Kret and Fischer, 2018). Since our ways of interpersonal interaction are changing, meaning that our cultural context is adapting, perhaps the way we perceive and recognize faces is evolving as well.

Also, in survival-related contexts, as the current pandemic situation might be, memory for survival-related information seems to be enhanced (Nairne et al., 2007). In this line of thought, exploring whether masked faces are perceived as more approachable because they are protected and do not represent a threat would be extremely valuable. While mandatory mask-wearing is disappearing in some countries, many parts of the world still require face masks, so understanding their impact on memory continues to be essential.

Future research adopting an eye-tracking paradigm would be of much interest to understand exactly what features of a masked face participants attend to. Such data would allow us a broader understanding of the face recognition phenomenon.

## Data availability statement

The raw data supporting the conclusions of this article will be made available by the authors, without undue reservation.

## Ethics statement

The studies involving human participants were reviewed and approved by Comissão de Ética em Investigação em Ciências Sociais e Humanas da Universidade do Minho. The patients/participants provided their written informed consent to participate in this study. Written informed consent was obtained from the individual(s) for the publication of any identifiable images or data included in this article.

## Author contributions

All authors listed have made a substantial, direct, and intellectual contribution to the work, and approved it for publication.

## Footnotes

1
https://osf.io/3vrdk/?view_only=39089853081142f78ad3df4984b7a5e8
